# MHQ: constructing an aggregate metric of population mental wellbeing

**DOI:** 10.1186/s12963-024-00336-y

**Published:** 2024-07-17

**Authors:** Jennifer Jane Newson, Oleksii Sukhoi, Tara C. Thiagarajan

**Affiliations:** Sapien Labs, 1201 Wilson Blvd, 27th floor, Arlington, Virginia USA

**Keywords:** Mental wellbeing, Population health, Mental health, Psychiatry, MHQ, Metric, Assessment, Productivity, Global mind project

## Abstract

**Background:**

According to the World Health Organization (WHO), mental health is ‘a state of wellbeing in which the individual realizes his or her own abilities, can cope with the normal stresses of life, can work productively and fruitfully, and is able to make a contribution to his or her community’. Any population metric of mental health and wellbeing should therefore not only reflect the presence or absence of mental challenges but also a person’s broad mental capacity and functioning across a range of cognitive, social, emotional and physical dimensions. However, while existing metrics of mental health typically emphasize ill health, existing metrics of wellbeing typically focus on happiness or life satisfaction, indirectly infer wellbeing from a selection of social and economic factors, or do not reflect a read out of the full spectrum of mental functioning that impacts people’s everyday life and that spans the continuum from distress and the inability to function, through to the ability to function to one’s full potential.

**Methods:**

We present the Mental Health Quotient, or MHQ, a population metric of mental wellbeing that comprehensively captures mental functioning, and examine how it relates to functional productivity. We describe the 47-item assessment and the life impact rating scale on which the MHQ metric is based, as well as the rationale behind each step of the nonlinear algorithm used to construct the MHQ metric.

**Results:**

We demonstrate a linear relationship between the MHQ metric and productive life function where movement on the scale from any point or in any direction relates to an equivalent shift in productive ability at the population level, a relationship that is not borne out using simple sum scores. We further show that this relationship is the same across all age groups. Finally, we demonstrate the potential for the types of insights arising from the MHQ metric, offering examples from the Global Mind Project, an initiative that aims to track and understand our evolving mental wellbeing, and since 2020 has collected responses from over 1 million individuals across 140 + countries.

**Conclusion:**

The MHQ is a metric of mental wellbeing that aligns with the WHO definition and is amenable to large scale population monitoring.

**Supplementary Information:**

The online version contains supplementary material available at 10.1186/s12963-024-00336-y.

## Background

The World Health Organization (WHO) defines mental health as ‘a state of wellbeing in which the individual realizes his or her own abilities, can cope with the normal stresses of life, can work productively and fruitfully, and is able to make a contribution to his or her community’ [1]. Mental health and one’s state of wellbeing is therefore not only determined by the absence of ill (mental) health, but also reflects a person’s broad mental capacity and functioning, such as their ability to be creative, achieve goals, take measured risks, form social relationships and regulate their emotions. To this end, a population metric of mental wellbeing that aligns with this definition can be constructed from a comprehensive evaluation of a broad spectrum of emotional, cognitive, physical and social functions of brain and mind that have an impact on people’s everyday life and that span the continuum from distress and the inability to function, through to the ability to function to one’s full potential. In this paper, we denote the term ‘mental wellbeing’ to specifically reflect this mental capacity and functioning that spans a continuum from negative to positive.

Several global and national metrics and indices of population mental health, happiness and wellbeing currently exist, such as the Institute for Health Metrics and Evaluation’s Global Burden of Disease [2], Gallups’s and the University of Oxford’s World Happiness Report [3], OECD’s Better Life Index [4] and Bhutan’s Gross National Happiness Index [5], with the development of new measures and metrics being an active area of research [6–10]. However, many of these existing approaches typically use single item measures of life satisfaction or happiness, or indirectly infer outcomes from a set of social factors such as income, education and healthcare. For example, the World Happiness Report determines ‘happiness’ by asking people to evaluate their present and future life using a Cantril Ladder scale ranging from 0 (worst possible life) to 10 (best possible life), while the OECD’s Better Life Index indirectly infers wellbeing through an evaluation of 11 domains (health, education, life satisfaction, housing, work-life balance, environment, jobs, safety, income, community, civic engagement). In contrast, Huppert and So [6] reviewed DSM-IV and ICD-10 symptom criteria for both anxiety and depression to identify 10 features of psychological well-being (competence, emotional stability, engagement, meaning, optimism, positive emotion, positive relationships, resilience, self-esteem, and vitality) by defining the opposite of common symptoms. This measure has been further developed into a multidimensional measure of subjective wellbeing (the WB-Pro) that includes 5 additional features (empathy, prosocial behavior, self-acceptance, clear thinking, and autonomy [7, 8]). However, while this measure offers a multidimensional metric of subjective wellbeing, it does not provide a read out of the full profile of cognitive, social, emotional and physical functioning of brain and mind that can impact everyday life. There is therefore an opportunity for a comprehensive construct of mental wellbeing that integrates the broad profile of cognitive, emotional, physical and social functioning required for a productive life that can be used effectively for tracking and understanding trends in mental wellbeing in the general population, as well as identifying potential drivers.

Accurately measuring and understanding the mental functioning of populations is critical in giving an accurate and real-time view of how people are faring and enables a deeper understanding of how changing social and environmental factors impact different facets of mental wellbeing. For instance, it could help explain why Finland has one of the highest suicide rates in western and northern Europe at 13 per 1000 [11] despite consistently having the highest ranking for life satisfaction, a term that is often interpreted and used interchangeably with happiness [3]. Data from such a metric is also particularly important in the context of current societal trends where mental health and wellbeing has declined to alarming levels over the past decade, particularly in younger generations [12–14]. Such a metric can enable understanding of evolving trends and show how various life experience, lifestyle and environmental factors differentially impact specific aspects of mental function. This can be used by researchers, clinicians, public health professionals and policy makers to guide the development and implementation of preventative strategies and solutions to improve mental wellbeing and monitor their magnitude of impact. Such approaches can also be implemented at various levels from governments through to organizations and establishments such as schools, universities and companies in the context of their students, employees and citizens.

One criteria for developing a metric of population mental wellbeing in this context is ensuring it is based on an assessment that captures the broad profile of mental functioning. Within the domain of mental health, a large number of measurement tools have been developed that typically focus on the symptoms of individual disorders, or take a cross-disorder perspective [15]. Within the domain of subjective wellbeing, numerous assessments also exist which include a wide variety of items relevant to subjective wellbeing [8–10, 16–20] and also share similarities with items assessed in mental health symptom questionnaires and interviews (e.g. energy, mood), albeit framed from a positive perspective. They also typically ask about other psychological perceptions such as those relating to life purpose, meaning, spirituality, as well as other life context perceptions (e.g. financial, safety) (see [20] for a review). However, these are indirect aspects of mental functioning and may be seen more as drivers or determinants of productive mental functioning rather than core aspects of mental functioning and therefore mental wellbeing itself.

**Fig. 1:**
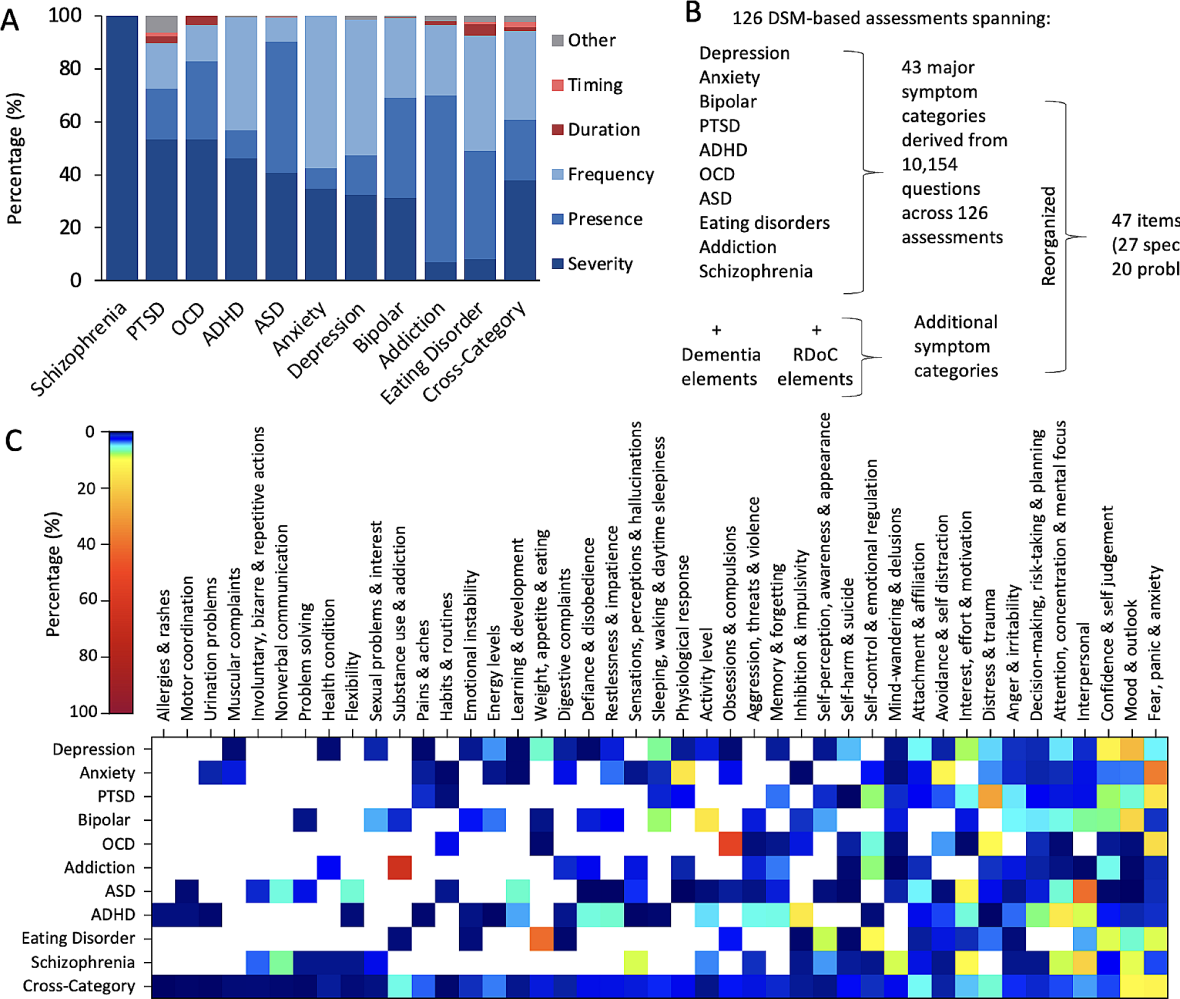
(**A**) The percentage of questions within each assessment tool, averaged across each disorder, which asked about the severity (dark blue), presence (mid blue), frequency (light blue), duration (dark red), timing (light red) of the symptom. (**B**) Diagram illustrating the method of development of the Mental Health Quotient. A total of 126 commonly used psychiatric assessment tools covering 10 disorders (as well as those taking a cross-disorder approach and elements from RDoc and dementia) were reviewed and consolidated into 43 symptom categories. ADHD: attention-deficit/hyperactivity disorder; ASD: autism spectrum disorder; DSM: Diagnostic and Statistical Manual of Mental Disorders; OCD: obsessive-compulsive disorder; PTSD: post-traumatic stress disorder; RDoC: Research Domain Criteria. (**C**) Representation of symptom categories across disorders. For each questionnaire or interview, the proportion of questions corresponding to each symptom category was calculated and averaged within a particular disorder to provide an aggregate view. Colours show the proportion (%) of questions from each of the 43 symptom categories for each disorder (averaged across assessment tools) and for cross disorder tools (white = 0%). Reproduced from [15]

A second criteria for a metric of population mental wellbeing in this context is that it should reflect a person’s ability to function, navigate adversities, and be productive in life. Therefore, it should not just capture “symptoms” or when something has gone “wrong” with a function, but also the positive aspects of a mental function (i.e. how functions can be an asset). Existing assessments of mental health disorders typically use a variety of scales that include the presence or absence of symptoms or estimates of their frequency, severity, or duration and can vary even within assessments of the same disorder grouping (Fig. 1A) [15], while scales within subjective wellbeing assessments also vary, but often use frequency or agree/disagree styled statements. However, these provide a unidimensional perspective of symptomatic or psychological experience that may not be equivalent in their life impact. For example, experiencing a symptom frequently but at a very low level of severity could have a lesser life impact than experiencing it rarely but with crippling severity.

In line with these criteria, we have previously described the development and validation of an assessment that comprehensively captures the broad profile of mental function and reflects life experience and consequence, from which an aggregate metric of population mental wellbeing could be constructed [21, 22]. For the development of this assessment, a comprehensive set of cognitive, social, emotional and physical functions were identified by categorizing 10,154 questions across 126 commonly used assessments spanning 10 major mental health disorders according to their functional and symptomatic characteristics [depression, anxiety, bipolar disorder, attention-deficit/hyperactivity disorder (ADHD), post-traumatic stress disorder (PTSD), obsessive-compulsive disorder (OCD), addiction, schizophrenia, eating disorder, and autism spectrum disorder (ASD), together with cross-disorder tools; Fig. 1B ] [15]. This gave rise to an initial list of 170 different subcategories of mental health symptoms and functions that were subsequently consolidated into a set of 43 categories by grouping together semantically similar subcategories in order to be as parsimonious as possible but yet comprehensive. The categorization of questions across the 126 assessments revealed a great deal of redundancy across disorder categories such that aggregating multiple disorder assessments into one would have substantial repetition. Importantly, none comprehensively captured all 43 categories and therefore were individually insufficient at assessing the full landscape of mental symptoms and functions [(Fig. 1C); see [15] for more details]. These 43 categories were subsequently reviewed in the context of other functional frameworks from neuroscience, [e.g., Research Domain Criteria, RDoC [23]] and neurology (e.g., dementia) and rearranged into a set of 47 semantically distinct items that were either problems or capacities that could be a challenge or an asset to one’s functioning (see Fig. 1B and Supplementary Table 1 for a full list of the 47 items and their descriptions) [22]. Other psychological perceptions that have also been associated with subjective wellbeing within the wider literature but are not mental functions (e.g. life meaning, purpose, financial, spiritual) are not included in this central construct, but are instead included as associated factors within a wider set of questions (see discussion).

**Fig. 2:**
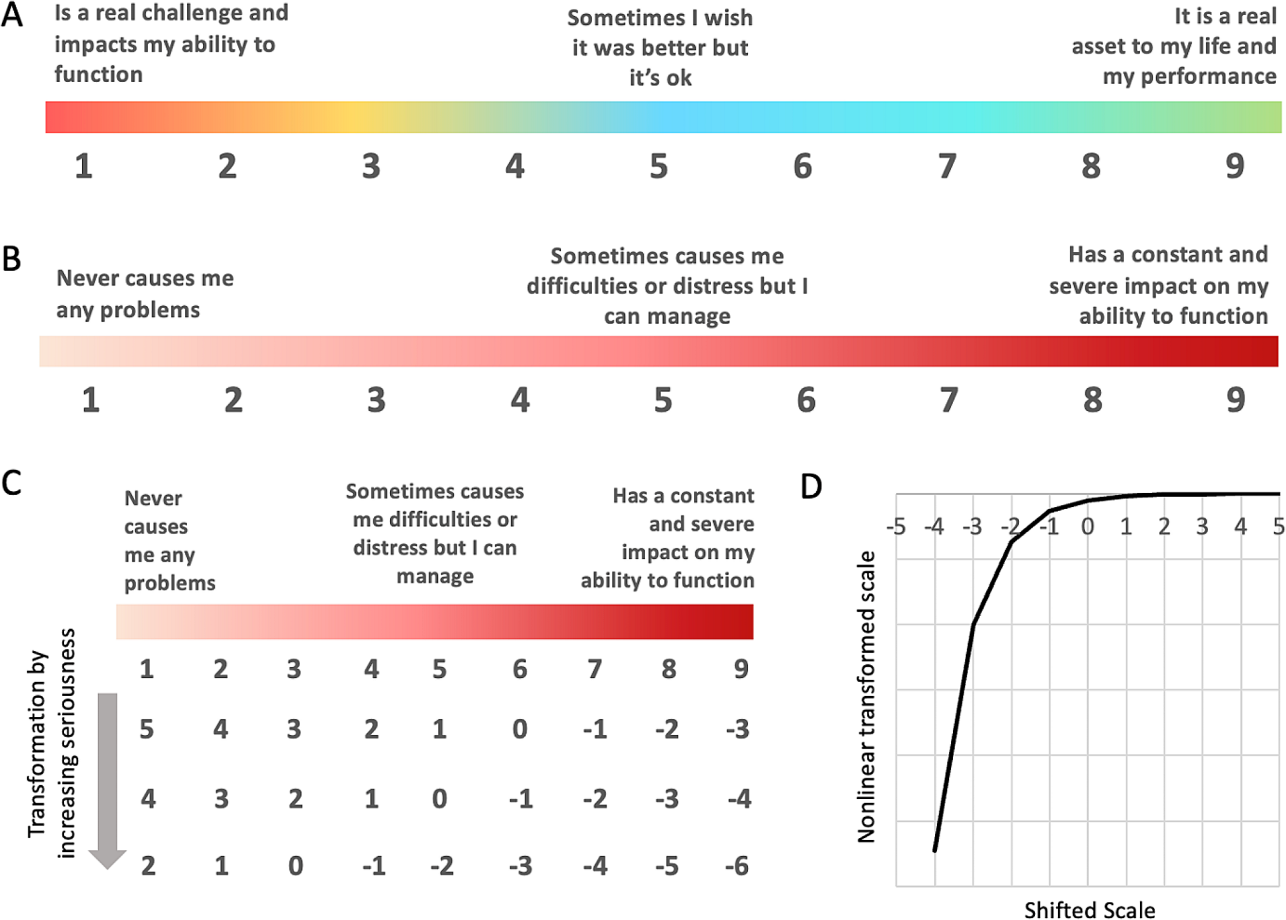
Illustration of the 1–9 life impact rating scale for spectrum (**A**) and problem (**B**) items (see methods). (**C**) An illustrative example for three tiers of increasing functional severity of problem items. (**D**) Nonlinear transformation of the scale that makes negative values more negative

The assessment evaluates these 47 problems and capacities using a scale that captures one’s current perception of their positive or negative life impact. This type of life impact scale therefore captures an integrated perspective of frequency, severity, and duration of any challenge that does not rely on recalled experience that can be difficult for a respondent to remember. Among these 47 items there are two categories, those aspects of mental function that exist on a spectrum from negative to positive function (spectrum items, for example, *Self-worth & confidence* and *Memory*), and those that are only negative problems (problem items, for example *Suicidal thoughts & intentions*). Two different life impact rating formats are therefore used within the assessment (Fig. 2A and B), both on a 9-point Likert scale. For the spectrum items (27 questions) 1 refers to ‘Is a real challenge and impacts my ability to function effectively’, 9 refers to ‘It is a real asset to my life and my performance’, and 5 refers to ‘Sometimes I wish it was better, but it’s ok’. In contrast, in the 9-point scale of problem items (20 questions) 1 refers to ‘Never causes me any problems’, 9 refers to ‘Has a constant and severe impact on my ability to function effectively’, and 5 refers to ‘Sometimes causes me difficulties or distress but I can manage’.

In this paper, we first describe the development of a novel aggregate population construct, or metric, of mental wellbeing called the Mental Health Quotient (MHQ) based on the assessment ratings of these 47 items, that aligns with the WHO definition above and reflects people’s mental capacity and functioning [1]. Many assessments that use a number-based rating scale simply compute an aggregate score as either the sum or average of raw scores across all questions (e.g., [10, 24]). However, this can result in individuals who are ‘middle of the road’ on all rated items having the same score as individuals who have several very severe problems in some areas and no problems in others. In addition, an individual with a small number of severe problems will have a ‘better’ score than an individual with a larger number of severe problems, although both may be equally incapacitated functionally. As an analogy within the domain of physical health, if rating scores on all physical problems were averaged, an individual whose only symptom was severe breathing difficulties would score more favorably than an individual with multiple moderate symptoms of fever, cough, cold and body ache. However, the individual with breathing difficulties may well be worse off functionally and have a higher probability of dying than the individual with multiple moderate symptoms. The same principle applies to mental health where functional capability is not necessarily about the number of symptoms, but about which symptoms they are, and their severity of consequence. The relevance and success of any scoring metric is therefore dependent on its ability to distinguish the more serious challenges from the less serious challenges.

We then describe how this aggregate metric distinguishes at risk individuals and relates linearly to functional productivity. Fundamentally, we sought to develop a metric that positions individuals on a continuum from distressed to thriving that was as close to linear as possible across the scale of function such that moving the same number of points in any direction from any place on the scale had a similar functional implication.

## Methods

### Demonstration of the MHQ scoring algorithm

#### Data sample

The data were taken from the Global Mind open-access database [25]. The sample included responses from 100,000 adults from 140 + countries collected between January 2023 and March 2023. Participants were recruited via outreach campaigns on Facebook and Google AdSense with advertisements containing the copy ‘Get your mental wellbeing score: Fast, Free, Anonymous’ along with a button linking to the start of the open survey. The advertisements were regionally targeted towards a series of age-sex groups between 18 and 85 + years using a broad range of interest keywords that had been optimized to ensure sufficient quotas in each age-sex group and broad geographic region. In addition, advertisements were continually and dynamically managed in response to feedback on the demographic composition of respondents to further ensure sufficient representation across age and biological sex groups. Starts and completions were tracked for each advertisement within each source (Google and Facebook) using Google and Facebook Analytics and data from all new sources were analyzed for parity before a new advertisement or source was scaled and included. The data were therefore from a non-probability sample of the internet-enabled population, with an unknown potential for sampling or non-response bias. However, trends from the Global Mind data for the United States have been shown to broadly mirror various trends of marital status, educational attainment and mental health treatment status acquired by the American Community Survey and Household Pulse Surveys conducted by the United States Census Bureau [26]. Biases in the representativeness of the data included a relatively small bias (~ 7%) towards single versus married respondents, 5–7% higher percentage of people not seeking treatment between the ages of 25 and 54, and lower percentage of people seeking treatment among the older age groups (4–5%). The demographic representativeness of samples from other countries is unknown.

All respondents completed the anonymous online MHQ assessment, providing ratings for the 47 items as well as answering questions on demographics and life experience factors [22]. Individuals took the assessment for the purpose of obtaining their personalized mental wellbeing report on completion. The provision of a personal report aimed to ensure the respondent answered the questions thoughtfully and accurately. MHQ scores were then calculated based on responses to the 47 items.

#### Computation of the MHQ Metric

The MHQ scoring algorithm is not computed as a simple average or sum of raw scores, but instead transformed in 3 steps, which includes (i) a threshold-based rescaling of the 9-point scale to a positive-negative scale, (ii) the application of a differential nonlinear weighting to negative scores to better distinguish at-risk populations, and (iii) a normalization of the scale into a window of -100 to + 200. Here we describe the 3 steps and the rationale behind each.

*Step 1: Categorizing items by severity and negative-positive thresholding*: First, the 47 items of the MHQ were categorized into three levels of functional severity based on their potential consequences to the individual or those around them. For example, *Suicidal thoughts or intentions* was categorized as having higher functional severity, while *Restlessness & hyperactivity* was considered as having lower functional severity. This means that on a 1–9 problem rating scale, *Suicidal thoughts or intentions* has a lower threshold (e.g. >4) at which rating values are considered negative compared to *Restlessness & hyperactivity* (e.g. >6). Due to directional differences in the spectrum and problem rating scales, this transformation is applied to problem items as “N – (rating response)”, while for spectrum items it is applied as “(rating response) – N”, where N = the level of functional severity. The specific values of N across the 47 items form part of a proprietary MHQ algorithm. Overall, this results in a shift of the life impact scale such that the 1–9 rating scale becomes a negative-positive scale where 0 is the threshold between negative and positive. Broadly, this threshold distinguishes those who are distressed or struggling at a level that requires intervention to help them function better (below 0) versus those who are simply managing normal ups and downs of life (above 0). An illustrative example for three tiers of problems is shown in Fig. 2C.

**Fig. 3:**
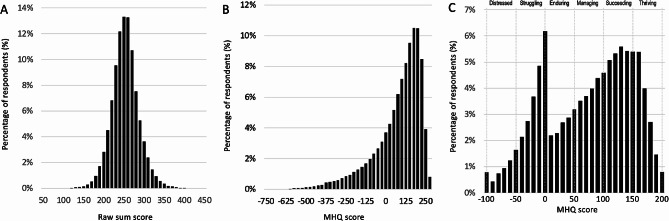
Comparison of sum scores and transformed sum scores. (**A**) Distribution of sum scores for 47 items across the whole population. (**B**) Distribution of transformed sum scores for 47 items across the whole population after thresholding and nonlinearly transforming the scale. (**C**) MHQ scores obtained after normalizing the negative and positive sides of the transformed sum scores, together with the MHQ score banding from distressed to thriving

*Step 2: 2: Nonlinear amplification of the scale*: Following this positive-negative thresholding, a nonlinear transformation is then applied to the scale to amplify the more negative scores and create greater distinction of at-risk groups by stretching out the negative side of the scale compared to the positive side (Fig. 2D). This transformation varies across the 47 items, and again was determined based on an evaluation of their functional severity, so that negative scores for items with higher functional severity become more negative than negative scores for items with lower functional severity. For example, a negative score of − 7 for *Suicidal thoughts or intentions* is amplified more negatively than a − 7 for *Restlessness and hyperactivity* and therefore contributes more negatively to the MHQ score. Similarly, a rescaled negative score of − 2 for *Energy levels* is amplified more negatively than a − 2 for *Creativity and problem solving* and contributes more negatively to the MHQ score. Following this transformation, the scores across the 47 items are summed such that individuals with negative scores on items with high functional severity are differentiated from those with negative scores for items with lower functional severity, even if their ratings for other items indicated they are doing ok for those items. As a consequence, the transformed distribution shifts from a normal distribution that you would observe if all ratings were simply summed together (sum scores; Fig. 3A), into a long-tailed distribution (Fig. 3B).

*Step 3: Normalizing the MHQ scale*: Following the creation of this long-tailed distribution to separate out individuals who are severely struggling with their mental wellbeing, we then normalize the scale to bring it back into a functional range. This serves two purposes, first to re-linearize the life impact and second to present scores that minimized any psychological distress that could be induced by receiving a highly negative score. This is accomplished by differently normalizing the negative and positive sides of the distribution so that the positive side of the scale ranges from 0 to 200 and the negative side ranges from − 1 to -100. Essentially, this compresses the long negative tail of the distribution to the left of the 0 value in the transformed distribution (Fig. 3B) so that 99% of individuals fall between − 1 and − 100 (Fig. 3C) with individuals within the remaining 1% placed within the − 100 group, resulting in a slightly higher prevalence in this group. The 99% value is used to normalize the negative scale (rather than 100%) because including this final 1%, which extends out far in the long-tailed distribution, compresses the majority of the data into too few score bins and reduces the resolution and linear range of the scores. For the purpose of interpretation, the scale is banded from distressed to thriving as shown in Fig. 3C.

The diagnosis of mental health disorders typically involves the presence of 5 or more symptoms associated with a particular disorder definition. To demonstrate how the MHQ algorithm separates out individuals who are severely struggling with their mental wellbeing, in Table 1 we show the distribution of the percentage of individuals with ≥ 5 severe symptoms [defined as MHQ items with either a rating of ≤ 2 for spectrum items or ≥ 8 for problem items] for each of 6 score groupings for sum scores and MHQ scores using data from the Global Mind Project collected during 2022 (see Application section below and [27] for more details). For sum scores, 80% of individuals were in the two mid-range score groups with 12% in the lower two score groups. In comparison, for the MHQ scores, only 36% of individuals were in the two mid- range score groups (Managing/ Enduring) while 63% were in the lower two score groups (Distressed/ Struggling). This demonstrates that those experiencing severe distress of some kind are more likely to be placed within the lowest score groups (Distressed/ Struggling) for MHQ scores compared to sum scores. In addition, as noted above, the MHQ algorithm preferentially classifies individuals experiencing those symptoms of a more severe nature (e.g. *Suicidal thoughts or intentions* or *Sense of being detached from reality*) into the Distressed/ Struggling score groups. Those with ≥ 5 symptoms who remain in the Enduring/ Managing score groups are therefore typically those experiencing symptoms of a lower functional severity (e.g. *Restlessness & hyperactivity*; *Sensory sensitivity*).

**Table 1:** Comparison of the number of respondents with ≥ 5 severe symptoms [≤ 2 rating (spectrum item) or ≥ 8 (problem items)] for sum scores and MHQ scores

Sum score group	Percentage distribution of people with ≤ 2 rating (spectrum item) or ≥ 8 (problem items) ratings for ≥ 5 items, *N* = 140,828	MHQ score group	Percentage distribution of people with ≤ 2 rating (spectrum item) or ≥ 8 (problem items) ratings for ≥ 5 items, *N* = 140,828
[47–109.7]	1.3	Distressed (<-50)	13.0
[109.7–172.3]	11.0	Struggling (-50 to < 0)	49.9
[172.3–235.0]	37.8	Enduring (0 to < 50)	23.5
[235.0–297.7]	41.8	Managing (50 to < 100)	12.5
[297.7–360.3]	8.0	Succeeding (100 to < 150)	1.2
[360.3–423.0]	0.1	Thriving (≥ 150)	0.0

### Validation of functional productivity

#### Data sample

Given that the primary criterion was to develop a score that was as linear as possible across the scale with respect to function, we examined functional productivity by asking 7,626 English-speaking respondents two additional questions within the MHQ assessment:

1. How many days during the past month were you able to work and carry out your normal activities, but could not get as much done because of problems with your physical or mental health? (Days unproductive)
2. How many days during the past month were you totally unable to work or carry out your normal activities because of problems with your physical or mental health (Days absent).

This data was obtained in September 2021. Respondents who completed the assessment in under 7 min (the minimum time needed to read all questions), took more than 60 min to complete the assessment, found the assessment difficult to understand (answered ‘No’ to the question: Did you find this assessment easy to understand?), or had responses with a standard deviation of less than 0.2 (representing people who answered with the same value across all 47 rating items) were excluded. This resulted in 7,377 responses (55% female, aged 18+) being availablefor the final analysis. We then analyzed the relationship between days unproductive and days absent and MHQ scores, as well as the simple sum of ratings across all items (sum scores) for an equal number of bins for both score types.

## Results

### The relationship between the MHQ score and functional productivity

**Fig. 4:**
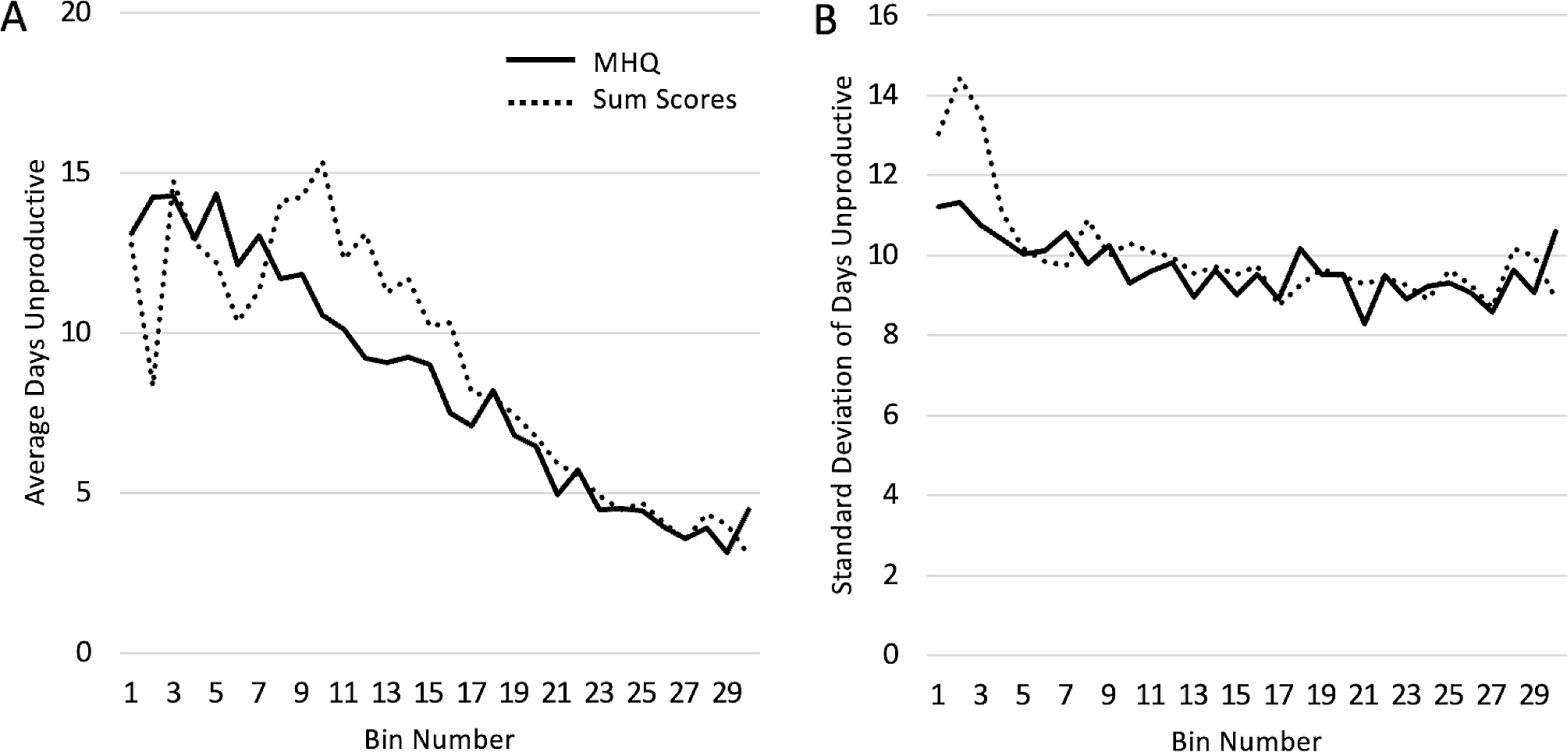
Average number of days unproductive (**A**) and corresponding standard deviation values (**B**) for each score bin for MHQ scores (solid line) and sum scores (dotted line)

Analysis of the relationship between MHQ scores and these independently acquired functional productivity responses showed that the average days unproductive changed linearly across the range of MHQ scores. Across the entire scale, the linear fit of population means had an R^2^ of 0.95 (*p* < 0.001). In contrast to the linear relationship with MHQ scores, days unproductive changed linearly only in the upper third of sum scores and was essentially flat across the lower third. Across the full range of sum scores, the linear fit of population means had an R^2^ of only 0.77. Thus, while a change of 10 MHQ points in any direction and at any point on the scale resulted in a similar functional change in terms of days unproductive, the bottom half of sum scores did not have any change in days unproductive (Fig. 4A; see Supplementary Table 2 for a statistical comparison between each bin). We note, however, that the standard deviation within each bin was similar between MHQ scores and sum scores except at the very lowest 5 bins, where sum scores had much higher standard deviation, indicating that there was much greater functional variability at this end of the scale for sum scores (Fig. 4B).

**Fig. 5:**
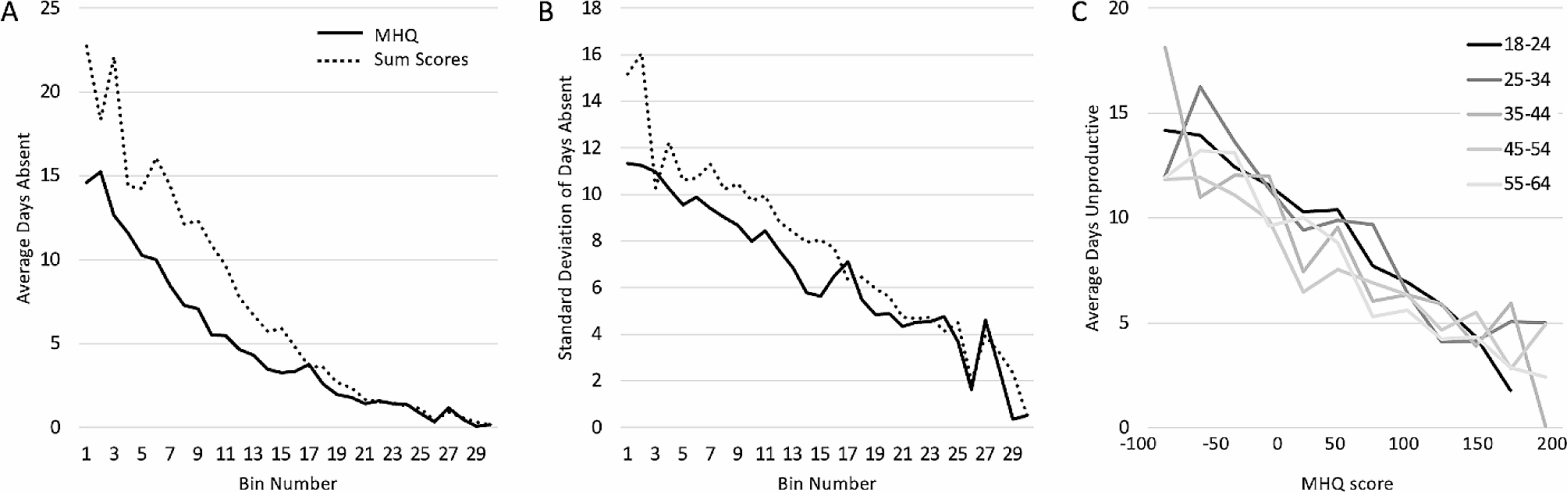
Average number of days absent (**A**) and corresponding standard deviation values (**B**) for each score bin for MHQ scores (solid line) and sum scores (dotted line). (**C**) Relationship between days unproductive and MHQ scores across different age groups

In contrast, days absent from work, which included absences due to both physical and mental health challenges, changed more exponentially than linearly for both sum scores as well as MHQ scores (Fig. 5A; see Supplementary Table 3 for a statistical comparison between each bin). However, the standard deviation of days absent within each bin, particularly in the bottom half of scores, was ~ 2 days lower for MHQ scores than for sum scores, showing that MHQ scores within each bin range were more functionally similar compared to sum scores (Fig. 5B). Thus, altogether, MHQ scores provide a metric of overall mental wellbeing that is a more reliable functional metric than sum scores.

We next looked at the relationship to productivity by age groups. One can imagine that different generations, or people at different stages in life, may evaluate the life impact of various mental functions differently based on their cultural perceptions and life experience. In addition, the specific symptoms that are dominant may vary by age. To determine if this relationship between productivity and mental wellbeing held constant by age, we plotted MHQ against days unproductive for each decadal grouping (Fig. 5C). For MHQ scores, the relationship with days unproductive was the same for each age group suggesting that, at an aggregate population level, the functional consequences of MHQ scores were comparable for all age groups. In contrast, sum scores were not only nonlinear but also highly variable across age groups in the upper third of the scale (Supplementary Fig. 1; note that the scale is reversed with higher scores which indicate higher problems on the left). Thus, altogether, shifts along the MHQ scale provide a more linear and consistent readout of productive function than sum scores for all adult age groups.

### Application of the MHQ assessment and metric

The MHQ assessment and metric are used within the Global Mind Project, an initiative that aims to track and understand our evolving mental wellbeing on a global scale and currently spans 140 + countries and 14 languages [28]. As of March 2024, the MHQ assessment had been taken by over 1.4 million people. In addition to providing a readout of the mental wellbeing of citizens across the world, the project also collects data on a broad range of demographic, lifestyle, and life experience factors that are used to provide a deeper understanding of the factors that promote or compromise people’s mental wellbeing. The inclusion of these factors also enable data samples to be described across multiple dimensions and constructed into representative samples that can be matched or weighted across geographies using commonly used descriptors such as age, biological sex or educational attainment. Beyond this, they also allow for the construction of more nuanced data samples that reflect the diversity of human populations across a wide variety of lifestyle and life experience factors (e.g. frequency of exercise, diet, childhood adversity & trauma).

Here we describe some of the results from this project that demonstrate the potential of this metric for tracking the evolution of mental wellbeing and identifying key drivers of population shifts.

#### Tracking mental wellbeing over time

**Fig. 6:**
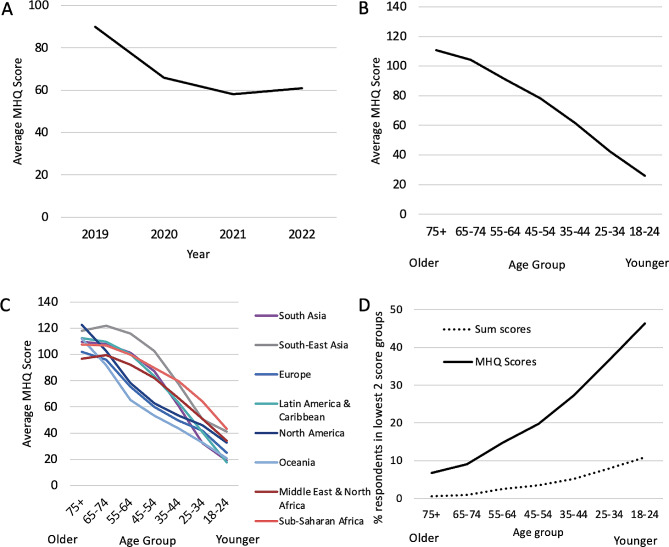
(**A**) Tracking changes in average MHQ score from 2019 to 2022 across 8 English-speaking countries. Error bars = ± SEM across countries. (**B**) Relationship between average MHQ score and age aggregated across all countries. Error bars = ± SEM across countries. (**C**) Relationship between average MHQ score and age across different geographic regions. Adapted from [27]. (**D**) Comparison of the percentage Distressed/Struggling for MHQ scores (black line), and the percentage two lowest sum score groups (dotted line), across age groups

The mental wellbeing of individuals and populations is not fixed, but instead varies over time in response to social and global factors. The Covid-19 pandemic was an example of a global event that had a substantial impact on population mental health as demonstrated by numerous studies documenting a rise in the prevalence of depression and anxiety [29–31]. However, while traditional diagnostic and assessment approaches track the rise in specific disorders or specific symptom combinations in line with clinical frameworks such as the DSM-5, these do not adequately capture people’s symptomatic experience which is highly heterogeneous, overlaps across multiple disorders, and changes over time [32–35]. Moreover, relying on assessment tools which only focus on clinical symptoms, precludes a holistic understanding of population mental wellbeing where individuals fall along a spectrum from distressed to thriving. While the Global Mind Project now collects data from over 140 countries, data collection began in 2019 from 8 English speaking countries. Computing the MHQ metric over time from these countries (see Supplementary Table 4 for N values and statistical comparisons between consecutive years) provides a unique holistic perspective on how population mental wellbeing has dynamically changed. To date, the results show that in the aggregate, MHQ scores dropped from an average of 90 ± 3.2 (SEM across countries) pre-pandemic (in 2019) to an average of 58 ± 1.7 in 2021, increasing only marginally to 61 ± 3.0 in 2022 (Fig. 6A) [27]. In productivity terms by using the equation of best fit, this translates to an aggregate decrease in productive days of ~ 2 per month per person from 2019 to 2022. Altogether, this gives an example of how the MHQ metric can be used to provide a perspective on how the mind of the world is changing and by inference, its productive capacity.

#### The decline in mental wellbeing across generations

Another major trend that has been documented, particularly in western countries where more epidemiological studies have been carried out, is an increase in rates of depression, anxiety and other mental health disorders in younger adults and youth [14, 36, 37]. However, this data has typically been fragmented due to methodological differences, with a focus on specific disorders or age groups. It is therefore not known how mental wellbeing has changed in the aggregate, nor how this shift looks across all age groups. If one were to aggregate all the epidemiological studies of various disorders it would still be substantially difficult, if not impossible, to determine the aggregate change in mental wellbeing given the substantial comorbidities and overlap of symptoms across disorders. While Global Mind data is not collected from youth under 18, we are able to examine the trend by age throughout adulthood using the MHQ metric. We show here that average MHQ scores decreased with each successively younger age group across the global sample (Fig. 6B; all comparisons between age groups: *p* < 0.001; t-test), with a similar pattern observed across multiple regions of the globe (Fig. 6C) [see [27] for further details]. We note that, at a population level, trends with average sum scores follow a similar pattern (Supplementary Fig. 2A, Supplementary Fig. 2B). However, using the bottom two bins of sum scores underestimated the percentage of individuals who were Distressed/Struggling (i.e. on average ≥ 5 severe symptoms) as these profiles were more widely distributed across the score range (Fig. 6D; Table 1).

#### Social trends and their relationship to mental wellbeing

**Fig. 7:**
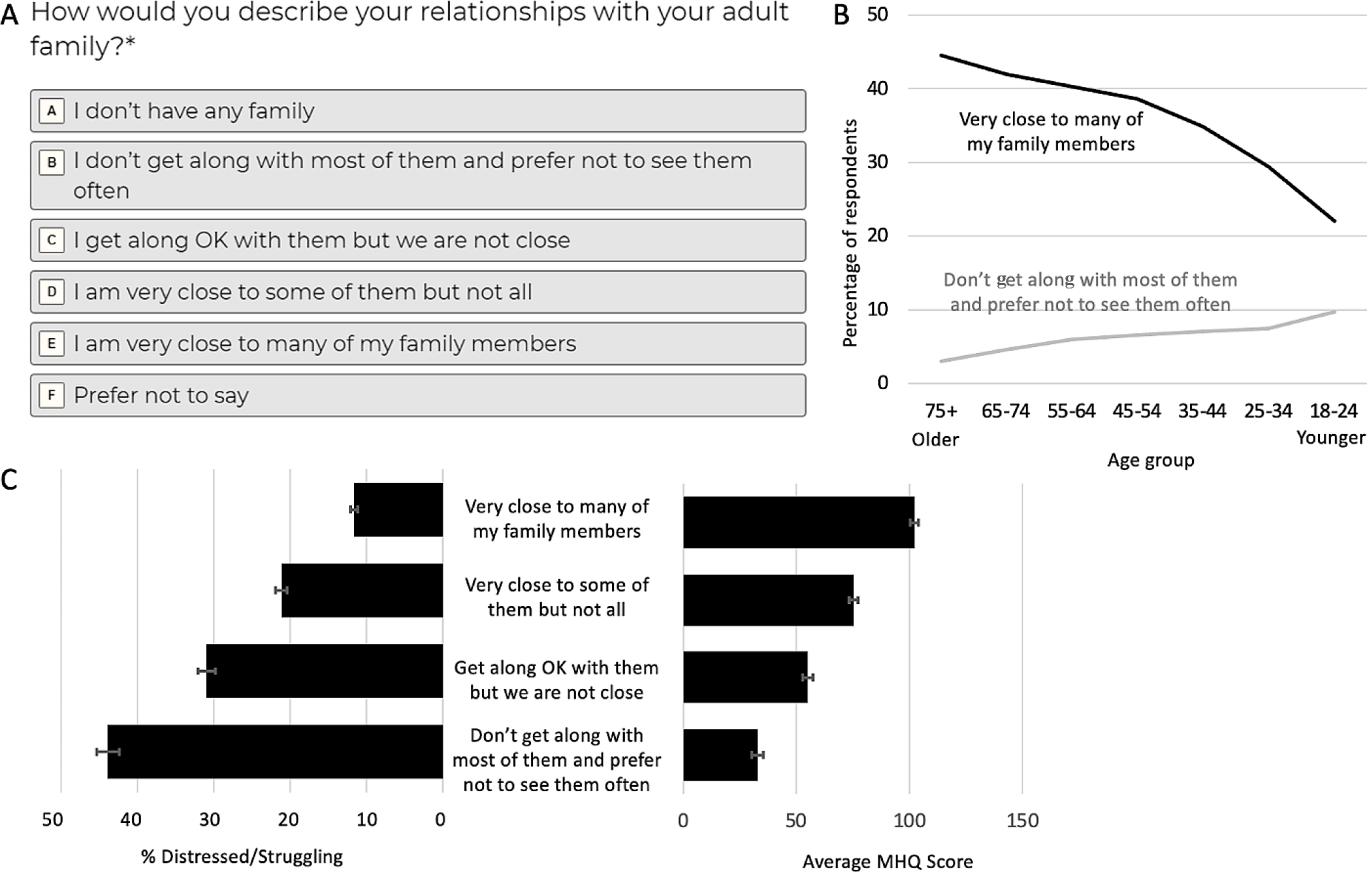
(**A**) Question about family relationships within the MHQ. (**B**) Percentage of responses (Error bars = ± SEM across countries) to the family relationship question across different age groups. (**C**) Relationship between family relationships and mental wellbeing across all respondents in the data sample. Left panel shows the % Distressed or Struggling (error bars show SEM across countries). Right panel shows the average MHQ scores (error bars show SEM across countries). Adapted from [27]

As a demonstration of the ability to use the MHQ assessment and metric to identify and quantify the relationship between different social trends and mental wellbeing, we provide the example of family relationships. Here we asked those who completed the MHQ assessment how close they were to their adult families (Fig. 7A). Across the globe, the percentage who reported being close to many members of their family decreased with each younger generation (Fig. 7B; N values and statistical comparisons shown in Supplementary Table 5). On average, only 22% of young adults aged 18–24 were close to their families compared to 44% of the oldest generation aged 75+ (*p* < 0.001), a two-fold difference. Conversely, 10% in the 18–24 age group did not get along with any of their family and preferred not to see them compared to only 3% of the oldest generation [*p* < 0.001; [27]]. Thus, the trend of generational decline in family relationships tracks the change in mental wellbeing described above.

We next examined mental wellbeing across all adults for each answer group. MHQ scores were highest for those who were close to many of their family members with an average of 102 ± 1.8 (SEM across countries), placed in the range we call “Succeeding”, and declining steadily to 33 ± 2.5 for those who did not get along with any of their family, in the range we call Enduring’ [*p* < 0.001; Fig. 7C; see [27] for more details]. Among those close to their families, 12% still struggled with their mental wellbeing (i.e., had MHQ scores < 0). However, this was almost four times lower than the 44% of those who did not get along with their families (*p* < 0.001). This 70 MHQ point difference and four-fold differential in mental wellbeing struggles was consistent across all age groups. This is a profound difference in risk, twice that of the mental wellbeing risks associated with other factors such as lack of exercise, lack of education or unemployment [38, 39].

While this does not prove definitively that deteriorating family relationships are the cause of poor mental wellbeing or vice versa, it demonstrates the ability to use the MHQ metric to identify relationships that can then be studied in more detail, and then validated in follow-up studies.

## Discussion

### Strengths of the MHQ metric

The MHQ metric has a number of strengths that are important to highlight. Firstly, it is based on an assessment derived from a comprehensive set of mental functions that spans 10 major mental health disorders as well as other neuroscientific and dementia frameworks. It therefore encompasses a holistic view of mental wellbeing that is more direct and comprehensive than other metrics that focus only on mental ill-health, are typically inferred from social factors or living conditions, or are based on single measures such as life satisfaction. Secondly, the assessment, although comprehensive, has been compiled in the most parsimonious manner possible, thereby enabling large-scale data acquisition by ensuring that assessment duration is not a barrier for completion. The MHQ metric therefore has the scope for application across large global populations. Thirdly, the metric is constructed using a life impact scale and nonlinear algorithm that results in a linear relationship to productive function across the entire scale, and that better distinguishes at risk individuals. This allows a functional interpretation of the score with practical life implications. Finally, the metric provides a perspective of the full spectrum of mental wellbeing from distressed to thriving such that it is possible to track subclinical changes in mental wellbeing that may not be immediately obvious in epidemiological studies that are based on traditional diagnostic criteria.

Overall, the MHQ metric is a novel measure of mental wellbeing that is a direct and comprehensive measure of mental capacity and functioning. Based on an assessment that is amenable to large scale data acquisition, it is therefore a unique tool for measuring and tracking the mental wellbeing of populations in various contexts. For instance, it can be used by schools, companies, and governments to provide a readout of how students, employees and citizens are faring, understand key drivers that can guide the development of targeted interventions, policies or strategies, and measure the impact of their implementation.

### Limitations of the MHQ metric

While the MHQ metric is based on a comprehensive assessment of mental functioning, one limitation is that no assessment that is amenable to ease of completion can capture all the nuances of mental health and wellbeing. In addition, mental health and wellbeing are multifaced concepts that span domains including psychiatry, psychology, neuroscience and public health. The MHQ was developed based on an analysis of 126 mental health assessments, spanning over 10,000 questions, but did not include assessments of subjective wellbeing, quality of life or personality traits, as it primarily focused on mental functioning and capacity. While there is considerable overlap in assessment items across these different domains, some items that are commonly associated with mental wellbeing or quality of life (e.g. life purpose, meaning, autonomy) are not included in the MHQ construct because they are not considered mental functions per se (see Supplementary Table 6 for a comparison of the MHQ and the WB-Pro [8]). However, they are included in the wider assessment of the Global Mind Project as relevant factors whose relationship to functional mental wellbeing can be assessed. In future, it would be useful to directly compare MHQ outcomes to wellbeing questionnaires such as the WB-Pro [8] or Warwick-Edinburgh Mental Wellbeing Scale (WEMWBS [10]) to determine how they compare. In addition, while the MHQ assessment touches on mental functions that could be considered personality traits (e.g. optimism) it does not comprehensively capture personality traits. However, there is a trade-off between the universe of functions, perceptions and personality traits and the ability to construct a practical assessment that is easy to complete.

A second limitation is that the metric arises from an assessment that utilizes online self-report. Since feeling is by its nature subjective, and there are no objective metrics (e.g., biomarkers) of feelings, any metric of mental wellbeing must rely on the self-report of these feelings. This is true of any assessment in the domains of psychiatry and psychology. It is therefore particularly important to benchmark self-reported ratings to more objectively measured functional outcomes. While being absent from work is a fairly objective metric, being unproductive is more subjective. In future, we plan to benchmark the MHQ metric against other objective measures of capability and productivity, such as testing of cognitive capability and tracking of time-use.

A third limitation is that the data are from a non-probability sample of the internet-enabled population, recruited via advertisements placed on Facebook and Google, with an unknown potential for sampling or non-response bias. However, the United States sample has been reported to be demographically similar to the United States national population [26]. The demographic representativeness of the samples from other countries is unknown.

Finally, as environmental circumstances and culture changes, it will be important to reassess the set of functions captured and their relationship to functional productivity. New mental functions and challenges may emerge in new environmental contexts as our expectations, the type of work we are required to do, and the tools that we have available to us, change. That said, such changes are unlikely to take place on the time scale of a few years but rather on a time scale of a decade or more.

In conclusion, we present the MHQ as a metric of mental wellbeing that reflects people’s mental capacity and functioning, aligns with the WHO definition [1], and is amenable to large scale population monitoring. Going beyond feelings of life satisfaction or happiness, it comprehensively captures 47 items of mental functioning to position individuals on a scale from distressed to thriving. Crucially, movement on the scale from any point or in any direction relates to an equivalent shift in productive ability.

### Electronic supplementary material

Below is the link to the electronic supplementary material.

Supplementary Material 1

Supplementary Material 2

## Data Availability

The dataset supporting the conclusions of this article is available in the Global Mind Project repository. Access can be requested here: https://sapienlabs.org/global-mind-project/researcher-hub/.
