# Supplementary material for: MHQ: constructing an aggregate metric of population mental wellbeing
Source: Popul Health Metr. 2024 Jul 17;22:16. doi: 10.1186/s12963-024-00336-y (PMC11256620; doi:10.1186/s12963-024-00336-y)

MHQ: Constructing an aggregate metric of population mental wellbeing

**Supplementary Figures**

Supplementary Figure 1: Relationship between days unproductive and sum scores across different age groups.


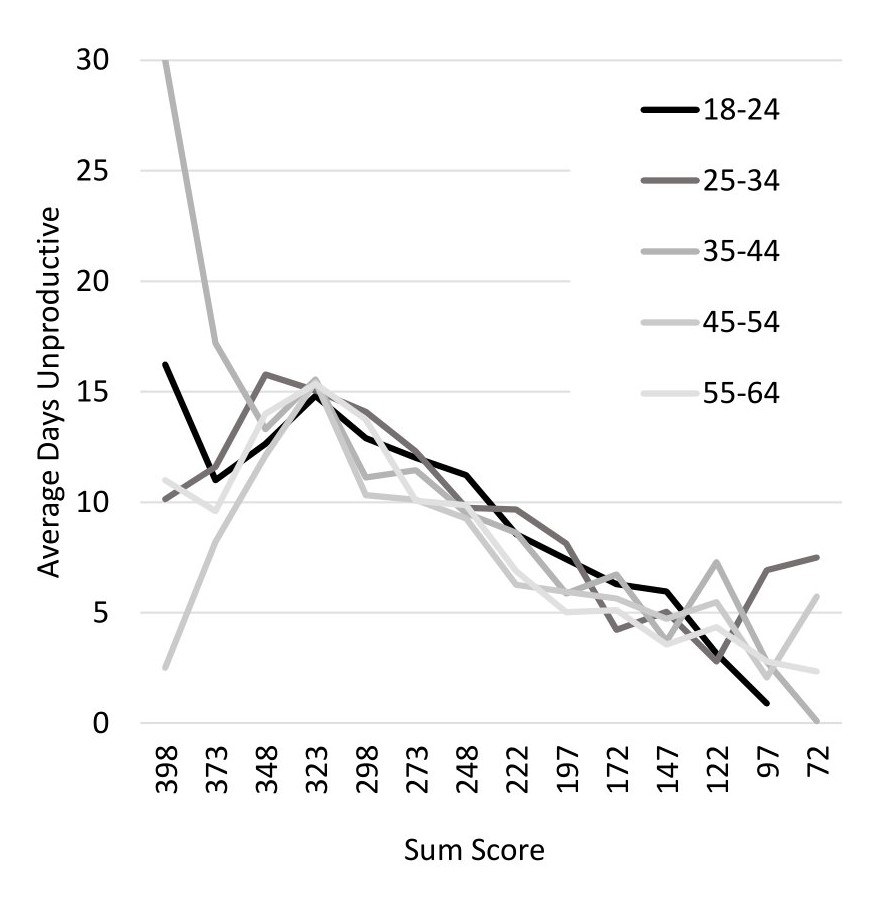


Supplementary Figure 2: Average sum scores across 8 English-speaking countries from 2019 to 2022 Error bars = ±SEM across countries. (B) Relationship between average sum score and age, aggregated across all countries. Error bars = ±SEM across countries.


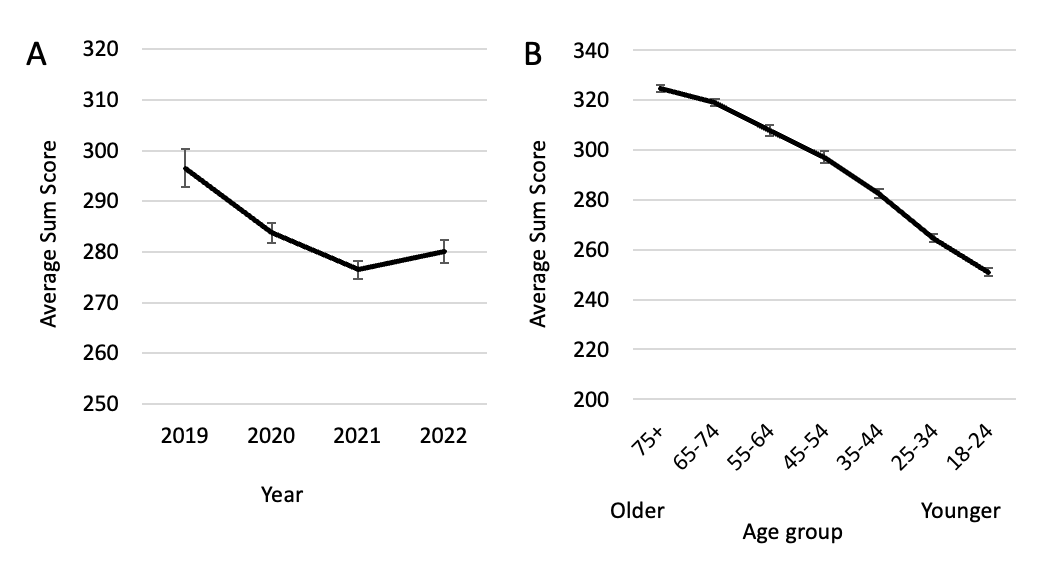

Supplement: Supplementary file 1 — Supplementary Material 1 [file 12963_2024_336_MOESM1_ESM.docx]
